# Activating SIRT3 in peritoneal mesothelial cells alleviates postsurgical peritoneal adhesion formation by decreasing oxidative stress and inhibiting the NLRP3 inflammasome

**DOI:** 10.1038/s12276-022-00848-3

**Published:** 2022-09-13

**Authors:** Tianli Shen, Yunhua Wu, Xingjie Wang, Zijun Wang, Enmeng Li, Cancan Zhou, Chenyang Yue, Zhengdong Jiang, Guangbing Wei, Jie Lian, Qinhong Xu, Xuqi Li

**Affiliations:** 1 Department of General Surgery, The First Affiliated Hospital of Xi’an Jiaotong University, 710061 Xi’an, Shaanxi China; 2 Department of General Surgery, Shaanxi Provincial People’s Hospital, 710061 Xi’an, Shaanxi China; 3 Department of Hepatobiliary Surgery, The First Affiliated Hospital of Xi’an Jiaotong University, 710061 Xi’an, Shaanxi China; 4 Department of Biology, York University, Toronto, ON Canada; 5 Department of Pathology, The First Affiliated Hospital of Xi’an Jiaotong University, 710061 Xi’an, Shaanxi China; 6 Department of Geriatric Surgery, The First Affiliated Hospital of Xi’an Jiaotong University, 710061 Xi’an, Shaanxi China; 7 Department of Talent Highland, The First Affiliated Hospital of Xi’an Jiaotong University, 710061 Xi’an, Shaanxi China

**Keywords:** Trauma, Molecularly targeted therapy, Acute inflammation

## Abstract

Peritoneal adhesions (PAs) are a serious complication of abdominal surgery and negatively affect the quality of life of millions of people worldwide. However, a clear molecular mechanism and a standard therapeutic strategy for PAs have not been established. Here, we developed a standardized method to mimic the pathological changes in PAs and found that sirtuin 3 (SIRT3) expression was severely decreased in adhesion tissues, which was consistent with our bioinformatics analysis and patient adhesion tissue analysis. Thus, we hypothesized that activating SIRT3 could alleviate postsurgical PAs. Sirt3-deficient (*Sirt3*^*−/−*^) mice exhibited many more PAs after standardized abdominal surgery. Furthermore, compared with wild-type (*Sirt3*^*+/+*^) mice, Sirt3-deficient (*Sirt3*^*−/−*^) mice showed more prominent reactive oxygen species (ROS) accumulation, increased levels of inflammatory factors, and exacerbated mitochondrial damage and fragmentation. In addition, we observed NLRP3 inflammasome activation in the adhesion tissues of *Sirt3*^*−/−*^ but, not *Sirt3*^*+/+*^ mice. Furthermore, mesothelial cells sorted from *Sirt3*^*−/−*^ mice exhibited impaired mitochondrial bioenergetics and redox homeostasis. Honokiol (HKL), a natural compound found in several species of the genus *Magnolia*, could activate SIRT3 in vitro. Then, we demonstrated that treatment with HKL could reduce oxidative stress and the levels of inflammatory factors and suppress NLRP3 activation in vivo, reducing the occurrence of postsurgical PAs. In vitro treatment with HKL also restored mitochondrial bioenergetics and promoted mesothelial cell viability under oxidative stress conditions. Taken together, our findings show that the rescue of SIRT3 by HKL may be a new therapeutic strategy to alleviate and block postsurgical PA formation.

## Surgery: Preventing abdominal scar tissue

Treatment with honokiol, a compound found in magnolia tree bark, significantly reduces formation of internal scar tissue after abdominal surgery in mice. Healing of incisions in the peritoneum, the connective tissue lining the abdomen, can result in scar tissue bonds known as peritoneal adhesions (PA), causing complications such as infertility or bowel obstructions. The mechanism of PA formation is unknown, and no therapies are available. Xuqi Li at The First Affiliated Hospital of Xi’an Jiaotong University, China, and co-workers found that PA tissues in both mice and human patients had decreased levels of SIRT3, a stress-response protein. Mice lacking SIRT3 showed increased inflammation and PA formation. When mice were treated with honokiol the day after surgery in order to boost SIRT3 levels, PA formation was significantly decreased. These results suggest a possible preventative treatment for post-surgical PAs.

## Introduction

Peritoneal adhesions (PAs) are common and severe postsurgical complications that result in abdominal pain, ileus, and female infertility and necessitate reoperation^1–4^. According to Mavros et al., the current morbidity rate of PAs is ~14.7%^5^. The National Institutes of Health estimates that PAs have a 20% rehospitalization rate and cause an extra 1 billion dollars in health care costs. Although the exact causes of PAs are still unclear, surgical trauma, ischemia, and inflammation have been reported to contribute to their formation^6^.

The peritoneum is a smooth, transparent, continuous membrane that lines the abdominopelvic cavity and covers some abdominal organs, such as the bladder, bowels, and spleen^7–9^. Mesothelial cells are located on the surface of the peritoneum; these cells provide a smooth, frictionless protective barrier and allow the movement of tissues and organs in the serous cavity^10–12^. Mesothelial cells help maintain microenvironment homeostasis by secreting molecules such as glycosaminoglycans, lubricants, and growth factors. Furthermore, mesothelial cells regulate fibrinolytic molecules to prevent PAs and have antigen presentation functions to mediate defense against infections^13–15^. When the peritoneum is injured, inflammation and coagulation are initiated, which leads to the formation of fibrin bands between adjacent surfaces^6,16^. DiZerega et al. clearly illustrated trends in the relative amounts and types of cells and fibrin deposits at sites of peritoneal damage^17^. The formation of adhesions can be divided into two stages. The early stage involves severe damage to mesothelial cells, the accumulation of reactive oxygen species (ROS), and the recruitment of neutrophils and lymphocytes^18^. The later stage involves fibrin deposition and fibrinolysis^6^.

Sirtuins are part of an evolutionarily conserved family that contains seven proteins with NAD^+^-dependent deacetylase activity in mammals, and SIRT3-SIRT5 are located primarily in mitochondria^19^. Shi et al. demonstrated that *Sirt3*^*−/−*^ mice are viable and metabolically unremarkable and exhibit normal body weights, oxygen consumption rates, respiratory exchange ratios, and activity levels; these findings are consistent with those of other studies demonstrating that *Sirt3*^*−/−*^ mice appear outwardly healthy under normal conditions and after mild stress, such as a 24 h period of starvation^20^. In contrast, some studies have revealed that *Sirt3*^*−/−*^ mice have ATP levels that are less than half those of wild-type mice and that *Sirt3*^*−/−*^ mice have a tendency to develop cardiac hypertrophy at a younger age than wild-type mice^19,21^. Similarly, SIRT3 levels are reduced in many mouse models of cancer, diabetes, acute kidney injury (AKI), and acute lung injury (ALI)^22–27^. However, whether SIRT3 levels are reduced in PAs is unknown.

Honokiol (3’,5-di-(2-propenyl)-1,1’-biphenyl-2,2’-diol, HKL) is a bioactive compound obtained from several species of the genus *Magnolia*^28^. Recent in vitro and in vivo studies have demonstrated that HKL has multiple biological activities, including anti-inflammatory, antithrombocytic, antiangiogenic, antitumor, antioxidative, and anticardiac hypertrophy effects^29–33^. Pillai et al. reported that HKL could be considered a natural SIRT3 activator that can directly bind SIRT3 and increase the enzymatic activity of SIRT3^29^.

Here, we reported that the expression of SIRT3 was impaired during PA formation, triggering the production of excess ROS and activating the NLRP3 inflammasome in adhesion tissues. Furthermore, we demonstrated that HKL, a candidate SIRT3 activator, may be a new therapeutic agent that alleviates ROS formation, inflammation, and NLRP3 activation to improve postsurgical PA formation.

## Materials and methods

### Experimental animals

Male C57BL/6 mice (aged 8–10 weeks) weighing 20–30 g were purchased from the Experimental Animal Center of Xi’an Jiaotong University, and *Sirt3*^*−/−*^ mice were obtained from the Jackson Laboratory. All procedures were approved by the Institutional Ethics Committee of Xi’an Jiaotong University and were conducted according to the Guidelines for the Institutional Animal Care and Use Committee of Xi’an Jiaotong University. All mice were maintained under specific conditions (24–25 °C, 50% humidity, and a 12 h:12 h day/night cycle) for 5 d before the experiments.

### Mouse PA model

**Fig. 1:**
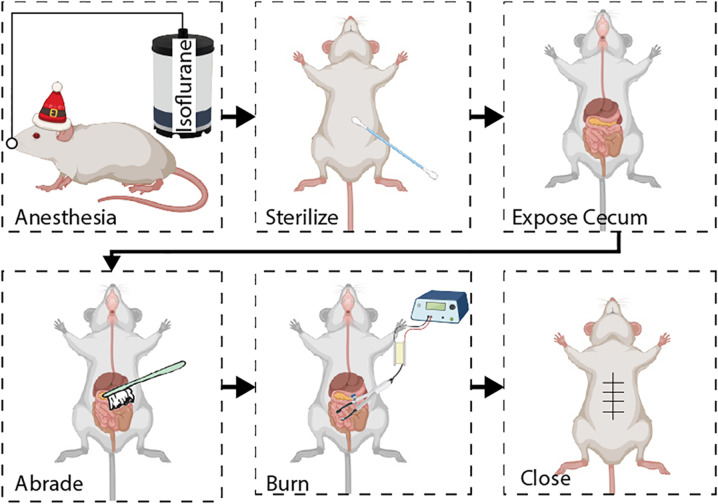
Flow chart of mouse PA model. Illustration showing the method used to establish a standardized model of postsurgical PAs in mice.

The mice were anesthetized with isoflurane until they were fully unconscious, as confirmed by the toe-pinch test. Each mouse was kept in the supine position on a sterile miniature operating platform. A heating pad was used to prevent anesthesia-induced hypothermia in mice throughout the procedure. The abdomen was cleaned with PBS and sterilized with betadine. During the procedure, the respiratory rate was monitored. An abdominal midline incision was made with a surgical scalpel. The underlying abdominal wall was then opened carefully, and the cecum was exposed. Next, the cecal wall was abraded 10 times with a small surgical brush. The parietal peritoneum along the abdominal wall was similarly abraded 10 times. Then, we cauterized the cecum to imitate hemostasis during bipolar electrosurgery. The cecum was then returned to its original anatomical position, and the abdomen was irrigated with prewarmed sterile PBS. The peritoneum was closed using 5-0 PGA sutures, and the skin incision was closed using 4-0 silk sutures. Buprenorphine was pipetted into the abdomen for postsurgical analgesia. The mice were monitored daily until they were euthanized. In the sham groups, the mice were treated in exactly the same way except that the incisions were closed without any abrasion or cauterization. An illustration of the method used to establish the PA model is shown in Fig. 1.

### Preparation of mouse primary mesothelial cells

Sham or adhesion peritoneal tissues were collected from the mice and minced on ice. The tissues were then digested for 2 h in a 37 °C shaker with 2 mg/mL collagenase (collagenase type II, Thermo Fisher), 0.5 mg/mL dispase (Dispase II, Thermo Fisher), and 2% fetal bovine serum (FBS; Gibco, Thermo Fisher) in RPMI 1640 medium (Gibco, Thermo Fisher) containing EDTA and HEPES. The digest was centrifuged at 500 × *g* for 15 min at 37 °C and resuspended in precooled PBS. Then, the digest was filtered through 90-, 70-, and 40 µm cell strainers (Corning). Red blood cell (RBC) lysis buffer (1×; eBioscience™ 1× RBC Lysis Buffer, Thermo Fisher) was used to lyse RBCs. The cells were counted and resuspended in FACS sorting buffer. The concentration was adjusted to 1 × 10^6^ cells/mL. The cells were then resuspended in RPMI 1640 medium containing 1% FBS and a 1:200 dilution of anti-CD16/32 Ab (Fc Block) and incubated for 1 h at 4 °C. Primary antibodies were added, and the cells were stained in the dark for 30 min at room temperature (RT). Primary mesothelial cells were defined as PDPN^+^LYVE1^−^CD31^−^CD45^−^ cells, immune cells were defined as CD45^+^CD31^−^ cells, endothelial cells were defined as CD31^+^CD45^−^ cells, and other cells were defined as CD45^−^CD31^−^LYVE1^+^ cells.

The antibody products used for FACS analysis of mesothelial cells included a FITC-conjugated rat anti-mouse CD45 (BD Pharmingen, 553080, used at 1:200), PE-conjugated hamster anti-mouse podoplanin (BD Pharmingen, 566390, used at 1:200), LYVE1 monoclonal antibodies (mAbs) (Thermo Fisher, 14-0443-82), PE-Cy™7-conjugated rat anti-mouse CD31 (BD Pharmingen, 561410, used at 1:200), APC-conjugated rat anti-mouse IgG1 secondary antibodies (eBioscience™, Thermo Fisher, 17-4015-82), purified rat IgG2a κ isotype control (BD Pharmingen, 553972, used at 1:200), and UltraComp eBeads™ Plus Compensation Beads (Thermo Fisher, 01-3333-41).

### Cell culture

Primary mesothelial cells were cultured in RPMI 1640 medium (Gibco) with 10% FBS (Gibco) in 96-well plates. HKL was purchased from Sigma‒Aldrich (H4914) and dissolved in DMSO. The human mesothelial cell line MeT-5A was kindly provided by Procell Life Science & Technology Co., Ltd. and was cultured in Medium 199 containing 10% FBS (Gibco), 3.3 nM EGF (Procell), and 400 nM hydrocortisone (Sigma‒Aldrich). All cells were maintained in a 5% CO_2_ incubator at 37 °C.

### Construction of stable SIRT3-knockdown cell lines

To generate stable SIRT3-knockdown MeT-5A cell lines, SIRT3^KD^-Puro lentiviruses (Suzhou GenePharma Co., Ltd.) were used to infect MeT-5A cells. After 72 h, stable SIRT3-knockdown cells were selected with 3 μg/mL puromycin (Sigma‒Aldrich).

### Measurement of cytokines

Adhesion tissues were homogenized with precooled PBS on ice and then centrifuged at 4 °C for 15 min at 12,000 × *g*. The levels of IL-6, IL-1β, and TNF-α were measured with ELISA kits (NanJing JianCheng Bioengineering Institute, China) according to the manufacturer’s instructions. TGFβ1 levels in adhesion tissues were detected with a TGFβ-1 Human/Mouse Uncoated ELISA Kit (Thermo Fisher).

### Measurement of oxidative stress in adhesion tissues

The concentrations of malondialdehyde (MDA), superoxide dismutase (SOD), and glutathione peroxidase (GPx) in PA tissues were measured as markers of oxidative stress. Adhesion tissues were homogenized and assessed using MDA, SOD, and GPx activity kits (NanJing JianCheng Bioengineering Institute, China).

### Immunofluorescence and immunohistochemical staining

Normal peritoneal and PA tissues were fixed with 4% PFA overnight at 4 °C. Then, the tissues were washed four times with precooled PBS, gently mounted in optimum cutting temperature (OCT) embedding compound, frozen at −20 °C, and then transferred to a −80 °C freezer. Tissue sections were prepared at a thickness of 5 µm and placed on histological slides. The sections were fixed in acetone for 12 min and then washed four times in PBS. Then, the sections were blocked and permeabilized with blocking buffer (2% BSA and 0.05% Triton-X in PBS) in a humidified chamber at RT for 1 h. The blocking buffer was drained from the slides, appropriate diluted primary antibodies were added, and the slides were incubated overnight at 4 °C. The slides were then washed three times with PBS and incubated with secondary antibodies for 1 h at RT. The slides were again washed three times with PBS, after which DAPI (ProLong™ Gold Antifade Mountant with DAPI, Thermo Fisher, P36935) was added, and the sections were covered with coverslips. For immunohistochemical staining, the tissues were visualized with horseradish peroxidase (HRP).

The following antibodies were used for immunofluorescence analysis: anti-NLRP3 (D4D8T) rabbit mAbs (Cell Signaling Technology, 15101), anti-cytokeratin 19 (Abcam, 52625), anti-SirT3 (D22A3) rabbit mAbs (Cell Signaling Technology, 5490), Alexa Fluor 594-conjugated goat anti-mouse IgG (H + L) cross-adsorbed secondary antibodies (Thermo Fisher, A-11005), Alexa Fluor 488-conjugated goat anti-rabbit IgG (H + L) cross-adsorbed secondary antibodies (Thermo Fisher, A-11005), Alexa Fluor 488-conjugated goat anti-rabbit IgG (H + L) cross-adsorbed secondary antibodies (Thermo Fisher, A-11008), and Alexa Fluor 594-conjugated goat anti-rabbit IgG (H + L) cross-adsorbed secondary antibodies (Thermo Fisher, A-11012). The antibodies/reagents used for immunohistochemistry were anti-α-smooth muscle actin (α-SMA) (Abcam, 5694), anti-collagen I (Abcam, 260043), and SignalStain® Boost IHC Detection Reagent (Cell Signaling Technology, 8114).

### Isolation of mitochondria

Sham/adhesion peritoneal mitochondria were isolated according to Frezza et al.^34^. Briefly, sham/adhesion tissues were minced into small pieces using scissors and washed three times with precooled PBS. The tissues were homogenized using a Teflon pestle and centrifuged at 1600 rpm. The homogenate was transferred to a 50 mL polypropylene Falcon tube and centrifuged at 700 × *g* for 15 min. Then, the supernatant was transferred to glass centrifuge tubes and centrifuged at 8000 rpm for 10 min. The pellet was resuspended in 5 mL of ice-cold mitochondrial isolation buffer, the cells were centrifuged at 8000 × *g* for 10 min, and the pellet containing mitochondria was resuspended. Finally, the mitochondria were stored in RIPA buffer at −80 °C.

### Mitochondrial membrane potential (Δψm) measurement

Primary mesothelial cells were plated in 96-well plates at a density of 2 × 10^4^ cells/well. The Δψm was measured using a Δψm assay kit (Beyotime Biotechnology, C2006) according to the standard instructions. Primary mesothelial cells were washed with PBS and incubated in JC-1 staining solution at 37 °C for 30 min in the dark. Then, the fluorescence was measured using a BD FACS Celesta™ flow cytometer.

### Hematoxylin and eosin (H&E) and picrosirius red (PSR) staining

Adhesion tissues were collected and fixed in 4% PFA for 24 h at 4 °C. Then, the tissues were embedded in paraffin, and 5 µm-thick sections were prepared. PSR solution (0.2%; Sigma‒Aldrich) or H&E was used for staining. Staining was evaluated and scored by three researchers (Tianli Shen, Yunhua Wu, and Zijun Wang) in a blinded manner using a microscope. The adhesion area was calculated using ImageJ 1.52k software.

### Western blot analysis

Equal amounts of protein were loaded and separated on SDS‒PAGE gels and transferred to nitrocellulose (NC) membranes (Thermo Fisher, IB23001). The membranes were blocked with 5% skim milk in 0.1% TBST and then incubated with primary antibodies at 4 °C for 12 h. The membranes were washed with 0.1% TBST three times and incubated with secondary antibodies for 1 h at RT. The signal was visualized using Pierce™ ECL Western blotting Substrate (Thermo Fisher, 32106). β-Actin was used as a loading control for total homogenates; Tom20 was used as a loading control for isolated mitochondria. The bands were quantified by densitometry using ImageJ 1.52k software.

The antibodies used for western blot analysis were as follows: anti-β-actin (Cell Signaling Technology, 4967), anti-Tom20 (D8T4N) rabbit mAbs (Cell Signaling Technology, 42406), anti-SirT3 (D22A3) rabbit mAbs (Cell Signaling Technology, 5490), anti-SOD2 (D9V9C) rabbit mAbs (Cell Signaling Technology, 13194), anti-cytochrome c (136F3) rabbit mAbs (Cell Signaling Technology, 4280), anti-NLRP3 (D4D8T) rabbit mAbs (Cell Signaling Technology, 15101), anti-cleaved caspase-1 rabbit mAbs (Cell Signaling Technology, 89332), anti-IL-1β (3A6) mouse mAbs (Cell Signaling Technology, 12242), anti-IL-18 (Abcam, 207323), and HRP-linked anti-rabbit IgG (Cell Signaling Technology, 7074).

### Real-time PCR analysis of mRNA levels

Total RNA was extracted from mouse adhesion tissues using TRIzol reagent (Invitrogen). Complementary DNA was synthesized with a Thermo Scientific RevertAid First Strand cDNA Synthesis Kit. Quantitative PCR was performed with iQ™ Supermix (Bio-Rad). The primer sequences are listed in Table 1.

**Table 1:** Sequences of the primers used for RT–PCR analysis.

Primer name	Species	Forward primer sequence (5′-3′)	Reverse primer sequence (3′-5′)
SIRT3	Mouse	ATCCCGGACTTCAGATCCCC	CAACATGAAAAAGGGCTTGGG
Collagen-1	Mouse	AAACCCGAGGTATGCTTGATCTGTA	GTCCCTCGACTCCTACATCTTCTGA
GAPDH	Mouse	TGAGGCCGGTGCTGAGTATGTCG	CCACAGTCTTCTGGGTGGCAGTG
α-SMA	Mouse	GAGGCACCACTGAACCCTAA	CATCTCCAGAGTCCAGCACA

### Oxygen consumption rate (OCR) measurement

A Seahorse XF96 system (Agilent) was used according to the manufacturer’s instructions. Briefly, mesothelial cells were plated in a 96-well plate at a density of 10,000 cells/well. The OCR was measured prior to treatment with 1.5 µM oligomycin, 0.5 µM FCCP, and 0.5 µM rotenone/antimycin A for three cycles.

### Measurement of ROS

ROS levels were measured using a general oxidative stress indicator (Thermo Fisher, C6827 and C10443) according to the manufacturer’s instructions. Primary mesothelial cells were plated in 96-well plates and treated with H_2_O_2_ (5 µM) in the presence or absence of HKL (5 µM) for 1 h. The cells were stained with the general oxidative stress indicator and measured with a BD FACS Celesta™ flow cytometer. The results were analyzed using FlowJo software.

### LIVE/DEAD staining

The viability of primary mesothelial cells was measured with a LIVE/DEAD Kit (Thermo Fisher, L3224). Briefly, primary mesothelial cells were plated in 96-well plates and treated with H_2_O_2_ in the presence or absence of HKL (5 µM) for 1 h. The cells were stained with calcein AM and ethidium homodimer-1. Fluorescence was measured with a fluorescence microscope (Leica Microsystems CMS).

### TUNEL staining

A TUNEL assay (Thermo Fisher, C10617) was used to examine apoptosis in PAs according to the manufacturer’s instructions.

### Human specimens

Human normal peritoneal tissues or PA tissues were obtained from patients who underwent abdominal surgery at the Department of General Surgery of the First Affiliated Hospital of Xi’an Jiaotong University. The experiments were approved by the Independent Ethics Committee of the First Affiliated Hospital of Xi’an Jiaotong University. Only tissues that would otherwise have been discarded were collected as specimens. For PA tissues, the patients were required to have a history of at least one abdominal operation and to have been diagnosed with postsurgical adhesions. For normal peritoneal tissues, the patients were required to have no history of abdominal operation or inflammation and no malignant diseases.

### Transmission electron microscopy

Mitochondria in adhesion tissues were evaluated using transmission electron microscopy (Hitachi, HT 7800, 120 kV). Adhesion tissues were fixed with glutaraldehyde, washed in cacodylate buffer, postfixed in 1% OsO_4_, dehydrated through ascending grades of alcohol, and finally embedded in Epon resin. The tissues were sliced using an ultramicrotome, and the ultrathin sections were mounted on copper grids and contrasted with 8% uranyl acetate and lead citrate. Mitochondria were observed with a Hitachi HT 7800 transmission electron microscope at 120 kV.

### Identification of differentially expressed genes (DEGs) and geneset enrichment analysis (GSEA)

DEGs between the sham and adhesion groups were identified by the R limma package and filtered by the following cutoff values: false discovery rate (FDR) < 0.05 and log2|FC | > 1. GSEA was performed using GSEA4.1.0 software, and c2.cp.kegg.v7.0.symbols.gmt in MSigDB was used as the reference geneset.

### Statistical analysis

The measurement data are expressed as the mean ± standard deviation (SD). Statistical analysis was performed using Student’s *t*-test (two-tailed), one-way ANOVA, or two-way ANOVA to evaluate differences between groups. Differences were considered statistically significant at *P* < 0.05. Analyses were performed using GraphPad Prism software (Mac version 8.4.1) or SPSS 18.0 software (SPSS Inc., USA).

## Results

### ROS accumulation and mitochondrial damage in adhesion tissues after peritoneal injury in mice

**Fig. 2:**
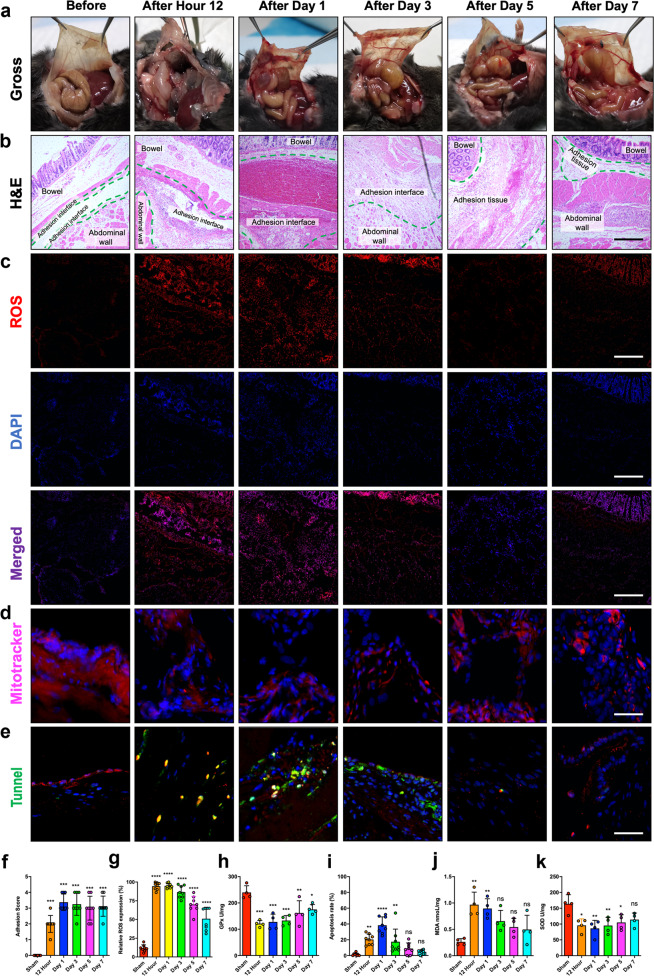
ROS accumulation and mitochondrial damage in adhesion tissues after peritoneal injury in mice. **a** Representative images showing the gross PA conditions 12 h, 1 d, 3 d, 5 d, and 7 d after peritoneal injury. **b** Representative H&E staining images of PA tissues 12 h, 1 d, 3 d, 5 d, and 7 d after peritoneal injury. Scale bars: 100 µm. **c** Representative images showing ROS accumulation (red fluorescence) in adhesion tissues 12 h, 1 d, 3 d, 5 d, and 7 d after peritoneal injury. Scale bars: 100 µm. **d** Representative images of mitochondria (magenta fluorescence) in adhesion tissues 12 h, 1 d, 3 d, 5 d, and 7 d after peritoneal injury. Scale bars: 100 µm. **e** Representative TUNEL staining images showing apoptotic cells in adhesion tissues 12 h, 1 d, 3 d, 5 d, and 7 d after peritoneal injury. Scale bars: 100 µm. **f** Adhesion scores based on Nair’s criteria at 12 h, 1 d, 3 d, 5 d, and 7 d after peritoneal injury. **g** Quantitation of ROS (red fluorescence) in adhesion tissues at 12 h, 1 d, 3 d, 5 d, and 7 d after peritoneal injury. **h**, **j**, **k** GPx, MDA, and SOD levels were evaluated in adhesion tissues. **i** Quantitation of apoptotic cells in adhesion tissues. The data are presented as the mean ± SD (*n* = 8). *P < 0.05, **P < 0.01, ***P < 0.001, ****P < 0.0001. One-way ANOVA.

To examine postoperative PA formation, we developed a mouse adhesion model that imitated the surgical procedure to the greatest extent possible and included three risk factors: (1) friction damage on the surface of the peritoneum, (2) peritoneal ischemia caused by compression, and (3) bipolar electrosurgery cauterization. The inclusion of all three risk factors generated a highly reproducible adhesion model in wild-type mice (Fig. 1). At 12 h, 1 d, 3 d, 5 d, and 7 d after peritoneal injury, the mice were euthanized, and their abdomens were carefully opened to observe the adhesion conditions and score the adhesions (Fig. 2a, b, f). Adhesions were visible as early as 12 h after surgery and appeared as soft attachments between organ surfaces (Fig. 2a, b). After one day, the adhesions had spread to other uninjured tissues, such as abdominal fat, the intestine, or the liver, and were characterized as hard/tough tissues. Furthermore, immunostaining showed that ROS rapidly increased by 12 h after surgery and peaked on the first day after surgery (Fig. 2c, d, g). In addition, the number of apoptotic cells increased at 12 h and peaked at 1 d, but apoptosis was not detected 5 d after the surgery (Fig. 2e, i). We also observed changes in some antioxidant markers, such as SOD activity and GPx levels. ELISA confirmed that the activity of both SOD and GPx was reduced in PA tissues after peritoneal injury (Fig. 2h, k). In contrast, ROS and MDA accumulated in adhesion tissues, which was consistent with the immunostaining results (Fig. 2g, j). These results suggest that peritoneal injury drives ROS accumulation and mitochondrial damage in PA tissues.

### SIRT3 expression is decreased in postsurgical adhesion tissues

**Fig. 3:**
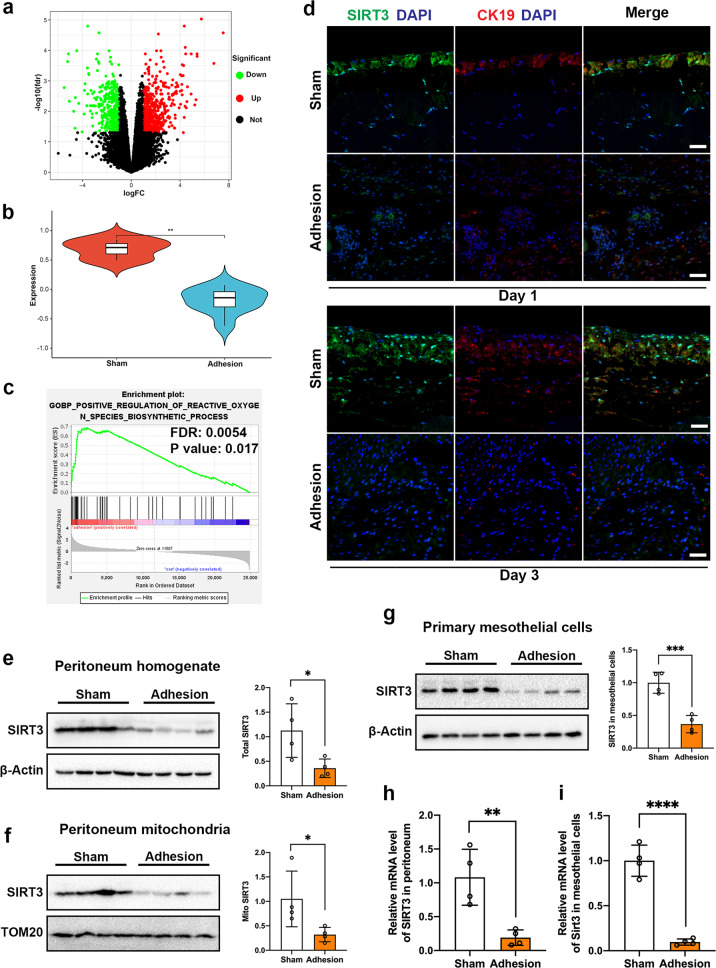
SIRT3 expression is decreased in postsurgical adhesion tissues. **a** Volcano map showing the distribution of DEGs in mouse cecal tissues between the postoperative adhesion group and sham operation group in the GSE123413 cohort. **b** The expression level of SIRT3 in the adhesion group and sham operation group in GSE123413. **c** GSEA enrichment curve showing the significant activation of positive regulation of reactive oxygen species biosynthetic processes in the postoperative adhesion group compared to the sham operation group. **d** Representative immunofluorescence images showing the expression of SIRT3 in sham and adhesion tissues. Scale bars: 100 µm. **e**–**g** Representative western blots showing SIRT3 in peritoneal tissue homogenate (**e**), peritoneal mitochondria (**f**), and primary mesothelial cells (**g**) from sham and adhesion tissues. **h, i** SIRT3 mRNA levels were measured in peritoneal tissue and primary mesothelial cells. The data are presented as the mean ± SD (*n* = 3–5). *P < 0.05, **P < 0.01, ***P < 0.001, ****P < 0.0001. Student’s *t*-test. All experiments were repeated three times.

SIRT3, which is a mitochondrial NAD^+^-dependent deacetylase in the sirtuin family, has been shown to correlate with several diseases, such as cardiac hypertrophy, acute kidney injury, and acute lung injury. The role of SIRT3 in postsurgical abdominal adhesion development is unclear. To determine the role of SIRT3 in postsurgical abdominal adhesion, we examined a GEO dataset, which provided RNA-seq data from the mouse adhesion and sham control groups. By comparing the RNA-seq data from the postsurgical adhesion groups with those from the sham groups, a total of 548 upregulated and 408 downregulated genes were identified in the adhesion groups compared to the sham groups, which were filtered by the following criteria: log_2_ | FC | > 1 and false discovery rate (FDR) < 0.05 (Fig. 3a). The expression levels of *Sirt3* were shown to be significantly downregulated in the adhesion groups compared with the sham groups, as shown in Fig. 3b and Supplementary Fig. 1. Moreover, the RNA expression levels of *Sirt* family genes (including *Sirt1-7*) in the sham and adhesion groups in the GSE123413 cohort are shown in Supplementary Fig. 2, and the expression levels of *Sirt3*, *Sirt4* and *Sirt5* were significantly decreased in the adhesion groups. Subsequently, geneset enrichment analysis (GSEA) was performed, and the results suggested that the positive regulation of reactive oxygen species (ROS) biosynthetic processes was significantly enriched in the adhesion groups (Fig. 3c). Initially, we examined whether the expression level of SIRT3 was altered in postsurgical PA tissues in mice. Immunostaining revealed that SIRT3 expression was lower 1 and 3 d after the operation in the adhesion groups than in the sham groups; CK19 (keratin 19) was stained as a specific marker of mesothelial cells (Fig. 3d). Consistent with the immunostaining results, western blot analysis confirmed that SIRT3 expression was decreased in whole peritoneal tissues and in isolated peritoneal mitochondria (Fig. 3e, f). To determine whether mesothelial cells are the predominant cell type in PA tissues, flow cytometry was used to analyze the proportions of mesothelial cells (PDPN^+^LYVE1^−^CD31^−^CD45^−^), immune cells (CD45^+^CD31^−^) and endothelial cells (CD31^+^CD45^−^) in PA tissues. The data showed that mesothelial cells were the predominant cell type in adhesion tissues at ~44.3% (39.3–49.2%) of total cells. Flow cytometry gating strategies are shown in Supplementary Fig. 3. To avoid interference from immune cells, endothelial cells, and other cells (CD45^−^CD31^−^LYVE1^+^), we digested the adhesion tissues and sorted primary mesothelial cells, immune cells, endothelial cells, and other cells using FACS. Western blot analysis confirmed that the reduction in SIRT3 expression was specific to mesothelial cells rather than immune cells, endothelial cells, or other cells (Fig. 3g and Supplementary Fig. 4). The decreased levels of *SIRT3* in mice with postsurgical adhesions were also evident at the mRNA level in whole peritoneal tissues and primary mesothelial cells (Fig. 3h, i). These results suggest that SIRT3 expression is decreased in postsurgical PAs.

### SIRT3 deficiency accelerates ROS formation and mitochondrial damage

**Fig. 4:**
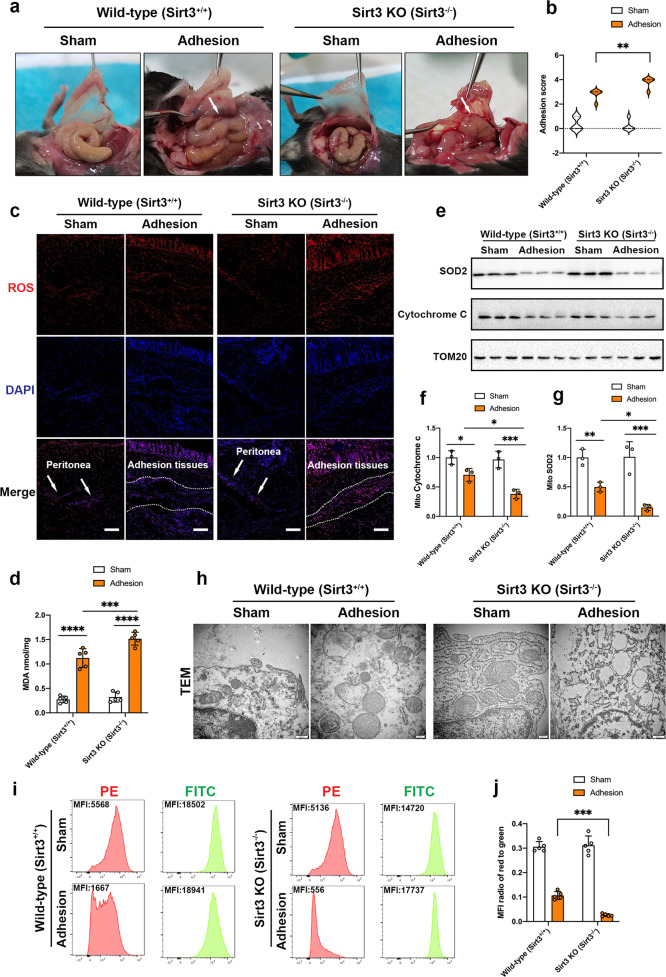
SIRT3 deficiency increases ROS formation and mitochondrial damage. **a** Representative images showing gross PA conditions in wild-type and *Sirt3*^*−/−*^ mice. White arrows indicate PAs. **b** Adhesion scores based on Nair’s criteria for wild-type and *Sirt3*^*−/−*^ mice. **c** Representative images showing ROS accumulation (red fluorescence) in adhesion tissues from wild-type and *Sirt3*^*−/−*^ mice. White arrows or white dashed lines indicate peritoneal or peritoneal adhesion tissues, respectively. Scale bars: 100 µm. **d** The levels of MDA in wild-type and *Sirt3*^*−/−*^ sham and adhesion tissues were determined by ELISA. **e**–**g** Representative western blots showing SOD2 and cytochrome c in the mitochondria of sham and adhesion tissues from wild-type and *Sirt3*^*−/−*^ mice. **h** Representative transmission electron microscopy images showing mitochondrial structure and swelling. Scale bars: 200 nm. **i**, **j** Representative flow cytometry images showing the Δψm in wild-type and *Sirt3*^*−/−*^ mesothelial cells. The data are presented as the mean ± SD (*n* = 3–5). *P < 0.05, **P < 0.01, ***P < 0.001, ****P < 0.0001. Two-way ANOVA. All experiments were repeated three times.

First, we determined whether SIRT3 deficiency exacerbated postsurgical PAs. The severity of adhesions was semi-quantified in wild-type and *Sirt3*^*−/−*^ mice according to the adhesion score criteria reported by Nair et al.^35^. Compared with wild-type mice, *Sirt3*^*−/−*^ mice exhibited exacerbated adhesion formation (Fig. 4a, b). Adhesion tissues were collected, and ROS production was measured using a ROS assay. Fluorescence images showed that ROS production in adhesion tissues was higher in *Sirt3*^*−/−*^ mice than in wild-type mice (Fig. 4c). To determine whether mesothelial cells were responsible for ROS accumulation, we sorted primary mesothelial cells from postsurgical adhesion tissues by FACS. ROS levels in live mesothelial cells were measured using CellROX oxidative stress reagents. The results showed that compared with those in wild-type mesothelial cells, higher levels of ROS accumulation were measured in *Sirt3*^*−/−*^ mesothelial cells (Supplementary Fig. 5). In addition, MDA, which is another useful marker of oxidative stress, was quantified using a lipid peroxidation assay kit, and the results showed that MDA levels in adhesion tissues were significantly higher in *Sirt3*^*−/−*^ mice than in wild-type mice (Fig. 4d). Since ROS can cause mitochondrial damage and fragmentation, we evaluated the expression of SOD2 and cytochrome c (cytochrome c oxidase), which are located in the mitochondria. Western blot analysis showed that the amounts of SOD2 and cytochrome c in mitochondria in adhesion tissues were more significantly decreased in *Sirt3*^*−/−*^ mice than in wild-type mice (Fig. 4e–g). Consistent with this finding, electron micrographs of PAs showed severe mitochondrial fragmentation into spheres and rarefaction in the tissues of *Sirt3*^*−/−*^ mice, while only slight swelling was observed in the tissues of wild-type mice (Fig. 4h). We also used JC-1 staining to monitor the Δψm of FACS-sorted mesothelial cells. Consistent with the electron micrograph data, mesothelial cells from *Sirt3*^*−/−*^ mice exhibited more robust mitochondrial depolarization than those from wild-type mice (Fig. 4i, j).

### SIRT3 deficiency exacerbates inflammation and NLRP3 activation

**Fig. 5:**
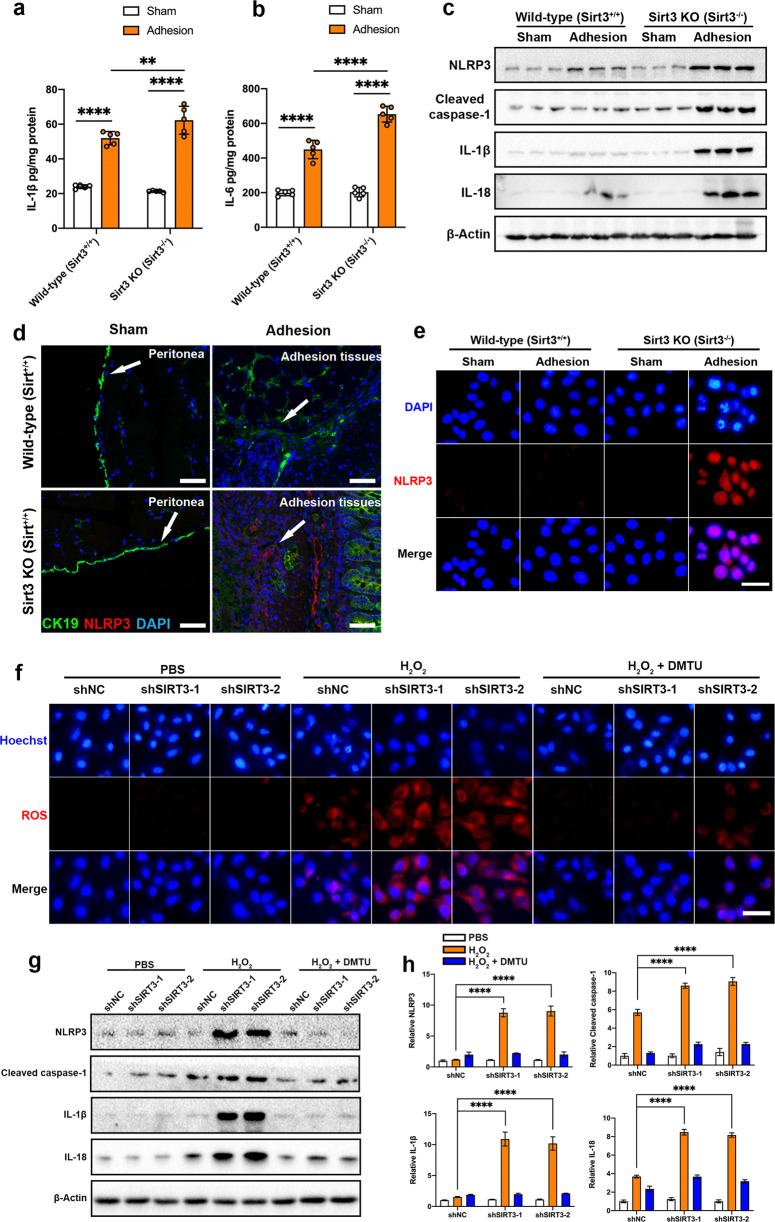
SIRT3 deficiency exacerbates inflammation and NLRP3 activation. **a**, **b** The levels of IL-1β and IL-6 in wild-type and *Sirt3*^*−/−*^ adhesion tissues were measured by ELISA. **c** Representative western blots showing NLRP3, cleaved caspase-1, IL-1β, and IL-18 in sham and adhesion tissues from wild-type and *Sirt3*^*−/−*^ mice. **d** Representative immunofluorescence images showing the expression of NLRP3 (red fluorescence) in sham and adhesion tissues from wild-type and *Sirt3*^*−/−*^ mice; CK19 (green fluorescence) was used as a marker of mesothelial cells. Scale bars: 50 µm. **e** Representative immunofluorescence images showing the expression of NLRP3 (red fluorescence) in sham and adhesion primary mesothelial cells from wild-type and *Sirt3*^*−/−*^ mice. Scale bars: 50 µm. **f** Representative fluorescence images showing the levels of ROS in shNC, shSIRT3-1, and shSIRT3-2 MeT-5A cells treated with H_2_O_2_ and/or DMTU. Scale bars: 50 µm. **g**, **h** Representative Western blots showing NLRP3, cleaved caspase-1, IL-1β, and IL-18 in shNC, shSIRT3-1, and shSIRT3-2 MeT-5A cells treated with H_2_O_2_ and/or DMTU. The data are presented as the mean ± SD (*n* = 3–5). **P < 0.01, ****P < 0.0001. Two-way ANOVA. All experiments were repeated three times.

Inflammation is a key factor in PA development. Therefore, we examined whether SIRT3 deficiency altered the levels of inflammation in the peritoneum after surgery. First, we compared the levels of three inflammatory cytokines in wild-type and *Sirt3*^*−/−*^ mice. The ELISA results showed that the levels of IL-1β, IL-6, and TNF-α were significantly higher in *Sirt3*^*−/−*^ mice than in wild-type mice (Fig. 5a, b and Supplementary Fig. 6). Furthermore, to confirm that these elevated levels of cytokines were due to mesothelial cells in adhesion tissues, we directly sorted mesothelial cells from adhesion tissues using FACS. The expression levels of IL-1β, IL-6, and TNF-α in *Sirt3*^*+/+*^ mesothelial cells and *Sirt3*^*−/−*^ mesothelial cells were measured by ELISA. As expected, the results showed that the levels of IL-1β and IL-6 were higher in *Sirt3*^*−/−*^ mesothelial cells than in *Sirt3*^*+/+*^ mesothelial cells (Supplementary Fig. 7). However, we could not detect a difference in TNF-α levels between the *Sirt3*^*−/−*^ mesothelial cells and the *Sirt3*^*+/+*^ mesothelial cells (Supplementary Fig. 7). Thus, these results suggest that mesothelial cells are the primary cells associated with IL-1β and IL-6 production. Moreover, mitochondrial dysfunction and the release of ROS and mtDNA into the cytoplasm are key events that activate the NLRP3 inflammasome. We found that NLRP3 was strongly activated in *Sirt3*^*−/−*^ adhesion tissues (Fig. 5c and Supplementary Fig. 8). We further measured the levels of cleaved caspase-1, IL-1β, and IL-18, which are downstream products of NLRP3. The data showed that the levels of cleaved caspase-1, IL-1β, and IL-18 were significantly increased in *Sirt3*^*−/−*^ mice (Fig. 5c and Supplementary Fig. 8). Consistent with the Western blot results, immunostaining showed that NLRP3 was activated in *Sirt3*^*−/−*^ adhesion tissues but not in wild-type adhesion tissues (Fig. 5d). Then, we sorted primary mesothelial cells from wild-type and *Sirt3*^*−/−*^ mice. Immunostaining confirmed that NLRP3 in mesothelial cells was activated to a greater extent in *Sirt3*^*−/−*^ mice than in wild-type mice (Fig. 5e and Supplementary Fig. 9). The activated NLRP3 inflammasome is a large protein complex that consists of a sensor (NLRP3), an adaptor (ASC) and an effector (Caspase-1)^36^. Activated caspase-1 cleaves and activates the proinflammatory cytokine IL-1β. To confirm that NLRP3 inflammasome activation did not increase NLRP3 protein expression, we measured NLRP3 protein expression using immunofluorescence (Fig. 5e and Supplementary Fig. 9), measured IL-1β levels in primary mesothelial cells using ELISA (Supplementary Fig. 7) and measured caspase-1 activity using the Caspase-Glo 1 Inflammasome Assay. The results showed that the caspase-1 activity was significantly increased in *Sirt3*^*−/−*^ mesothelial cells (Supplementary Fig. 10). Taken together, these results suggest that the NLRP3 inflammasome is highly activated in mesothelial cells sorted from peritoneal adhesion tissues of *Sirt3*^*−/−*^ mice.

To investigate whether SIRT3 deficiency activated NLRP3 and upregulated IL-1β expression in vitro, we treated the human mesothelial cell line MeT-5A with hydrogen peroxide to mimic the accumulation of ROS in mesothelial cells during PA formation (Fig. 5f). shSIRT3 lentiviruses were used to stably knock down SIRT3 expression, which was further confirmed by western blotting and PCR (Supplementary Fig. 11). The results showed that hydrogen peroxide treatment upregulated the expression of NLRP3, caspase-1, IL-1β, and IL-18 in shSIRT3-MeT-5A cells compared with shNC-MeT-5A cells (Fig. 5g, h). Of note, scavenging ROS with N,N’-dimethylthiourea (DMTU) rescued the expression of NLRP3, caspase-1, IL-1β, and IL-18 in shSIRT3-MeT-5A cells (Fig. 5g, h). Thus, we determined that SIRT3 deficiency caused NLRP3 inflammasome activation in MeT-5A cells and primary mesothelial cells sorted from peritoneal adhesion tissues of *Sirt3*^*−/−*^ mice.

### Honokiol reduces ROS formation and NLRP3-related inflammation

**Fig. 6:**
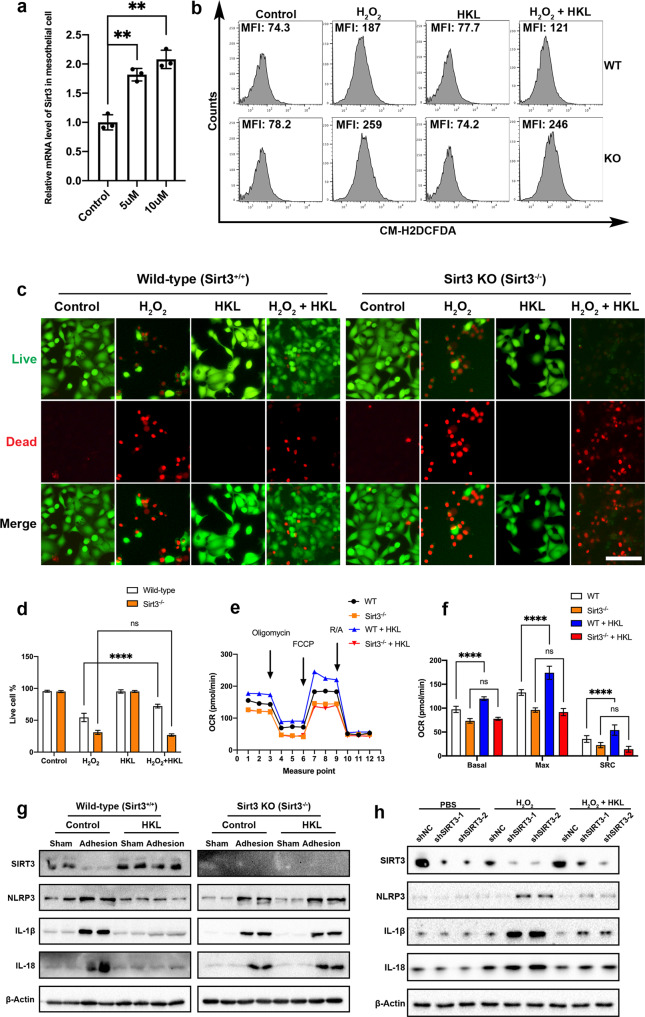
HKL reduces ROS formation and NLRP3-related inflammation. **a** SIRT3 mRNA levels after treatment with HKL (5 µM and 10 µM) for 6 h. **b** Representative flow cytometry images showing the levels of ROS in wild-type and *Sirt3*^*−/−*^ mesothelial cells treated with H_2_O_2_ and/or HKL. **c**, **d** Representative LIVE/DEAD staining images showing cell viability after treatment with H_2_O_2_ (5 µM) and/or HKL (5 µM). Scale bars: 50 µm. **e** Representative traces showing the OCRs in wild-type and *Sirt3*^*−/−*^ mesothelial cells treated with HKL. FCCP carbonyl cyanide-4-(trifluoromethoxy)phenylhydrazone, R/A rotenone/antimycin. **f** Quantitation of the OCRs in wild-type and *Sirt3*^*−/−*^ mesothelial cells treated with HKL. Basal basal OCR, Max maximal OCR, SRC spare respiratory capacity. **g** Representative Western blots showing SIRT3, NLRP3, IL-1β, and IL-18 in sham and adhesion tissues from wild-type and *Sirt3*^*−/−*^ mice treated with Intralipid or HKL. **h** Representative western blots showing SIRT3, NLRP3, IL-1β, and IL-18 in shNC, shSIRT3-1, and shSIRT3-2 MeT-5A cells treated with H_2_O_2_ and/or HKL. The data are presented as the mean ± SD (*n* = 4–5). ns not significant, *P < 0.05, ****P < 0.0001. One-way ANOVA or two-way ANOVA. All experiments were repeated three times.

Honokiol (HKL), a small-molecular-weight natural biphenolic organic compound, is extracted from the bark of magnolias^28^. We found that HKL increased the mRNA level of *SIRT3* in primary mesothelial cells in a dose-dependent manner (Fig. 6a). To determine whether HKL can reduce ROS production in mesothelial cells, wild-type mesothelial cells and *Sirt3*^*−/−*^ mesothelial cells were treated with 5 µM H_2_O_2_ in the presence or absence of HKL (5 µM). FACS analysis showed that HKL treatment significantly reduced H_2_O_2_-induced ROS levels in wild-type mesothelial cells but only slightly reduced ROS levels in *Sirt3*^*−/−*^ mesothelial cells (Fig. 6b and Supplementary Fig. 12). Furthermore, to assess the ability of SIRT3 to rescue cells from ROS-induced cell death, LIVE/DEAD staining was performed. Consistent with the ROS results, the cell death assay results showed that HKL treatment rescued wild-type mesothelial cells but not *Sirt3*^*−/−*^ mesothelial cells (Fig. 6c, d). Next, the ability of SIRT3 to alter mitochondrial bioenergetics was assessed. The OCR results showed that wild-type mesothelial cells exhibited higher OCRs than *Sirt3*^*−/−*^ mesothelial cells, and HKL treatment evidently increased the OCRs of wild-type mesothelial cells but not *Sirt3*^*−/−*^ mesothelial cells (Fig. 6e, f). Based on the beneficial effects of HKL on primary mesothelial cells in terms of mitochondrial bioenergetics and ROS-induced cell death, we next examined whether HKL could reduce inflammation in postsurgical abdominal adhesions. Mice were treated with 0.2 mg HKL/kg/d in 20% Intralipid (Sigma‒Aldrich) once daily for 3 consecutive days. Western blot analysis of PAs revealed that HKL treatment suppressed NLRP3 inflammasome activation in wild-type mice but not in *Sirt3*^*−/−*^ mice. Moreover, the levels of the NLRP3-related inflammatory factors IL-1β and IL-18 were reduced in wild-type mice (Fig. 6g and Supplementary Fig. 13). To investigate whether HKL treatment could suppress NLRP3 inflammasome activation and reduce IL-1β and IL-18 in vitro, shNC MeT-5A cells and shSIRT3 MeT-5A cells were treated with 5 µM H_2_O_2_ in the presence or absence of HKL (5 µM). The results showed that HKL treatment suppressed the NLRP3 inflammasome in shNC MeT-5A cells and reduced IL-1β and IL-18 expression levels in both shNC and shSIRT3 MeT-5A cells (Fig. 6h and Supplementary Fig. 14). These results suggest that HKL reduces ROS formation and NLRP3-related inflammation in vitro and in vivo.

### Immediate administration of HKL reduces the occurrence of postsurgical PAs

**Fig. 7:**
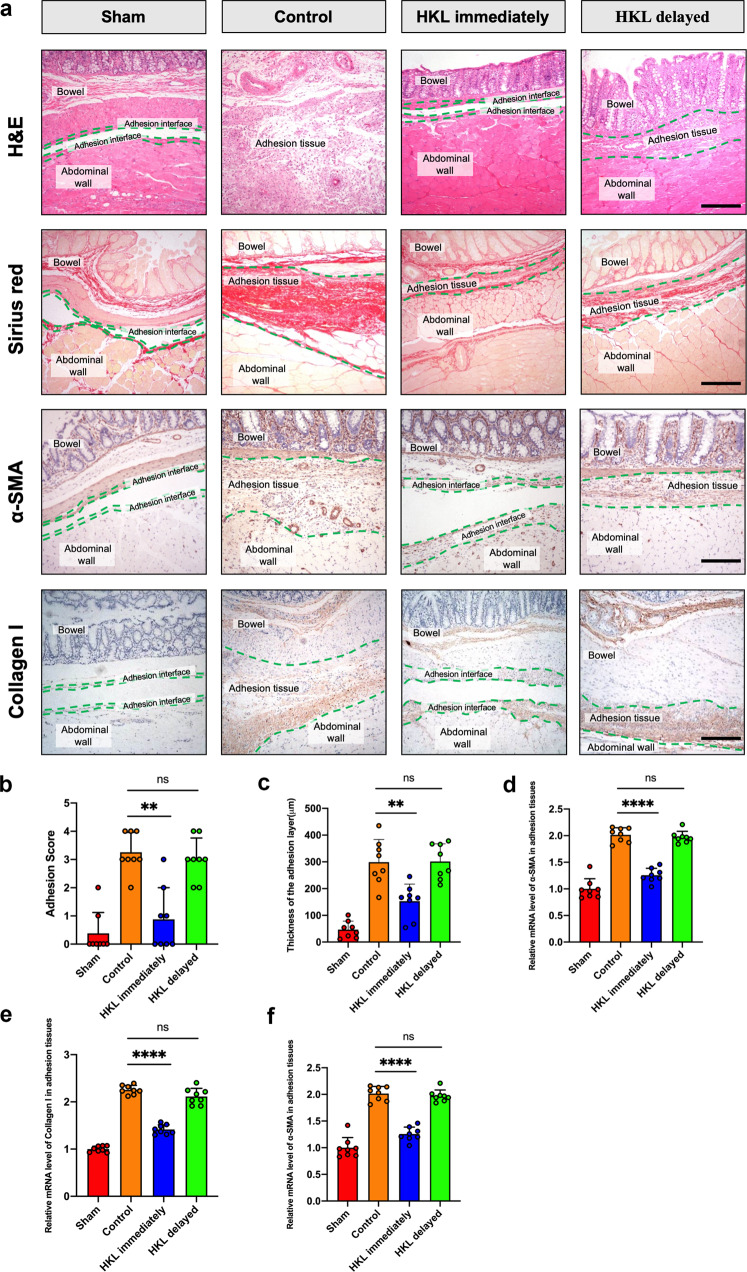
HKL reduces the occurrence of postsurgical PAs. **a** Representative images showing PA conditions immediately after treatment with Intralipid, immediate HKL, or delayed HKL; picrosirius red (PSR) staining images indicating the thickness of the adhesion layers. Immunohistochemical staining images showing the expression of collagen I and α-SMA in adhesion tissues. Scale bars: 200 µm. **b** Adhesion scores based on Nair’s criteria after treatment with Intralipid, immediate HKL, or delayed HKL. **c** Thickness of the adhesion layers after treatment with Intralipid, immediate HKL, or delayed HKL. **d**, **e** The mRNA levels of collagen I and α-SMA in adhesion tissues. **f** The levels of TGF-β in adhesion tissues were measured by ELISA. The data are presented as the mean ± SD (*n* = 8). ns not significant, **P < 0.01, ****P < 0.0001. One-way ANOVA.

Fiber and collagen formation occurs during the last stage of postoperative PA development as a means of repair after peritoneal injury. To investigate whether pretreatment with HKL could reduce collagen formation, we randomly divided the mice into three groups: a control group, in which mice received 20% Intralipid once daily for 3 d starting on the first day after surgery; an immediate-HKL group, in which mice received 0.2 mg HKL/kg/d in 20% Intralipid once daily for 3 d starting on the first day after surgery; and a delayed-HKL group, in which mice received 0.2 mg HKL/kg/day in 20% Intralipid once daily for 3 d starting on the fourth day after surgery. H&E staining showed that immediate-HKL group mice exhibited milder adhesion formation and received significantly lower scores than control group mice (Fig. 7a, b). However, adhesions in delayed-HKL group mice were not milder than those in control group mice. Picrosirius red (PSR) staining showed that the adhesion layers were thinner in the immediate-HKL group than in the control group (Fig. 7a, c). Immunohistochemistry showed that the expression of α-SMA and collagen I was significantly reduced in adhesion tissues, which was consistent with the difference observed at the mRNA level (Fig. 7a, d, e). TGF-β is considered to be an important factor in PA development that regulates mesothelial-mesenchymal transition. Thus, we measured TGF-β levels and found that they were decreased when HKL treatment was applied immediately after surgery (Fig. 7f). Taken together, these findings indicate that the immediate application of HKL is beneficial in reducing the occurrence of peritoneal adhesions.

### SIRT3 expression is downregulated in human PA tissues

**Fig. 8:**
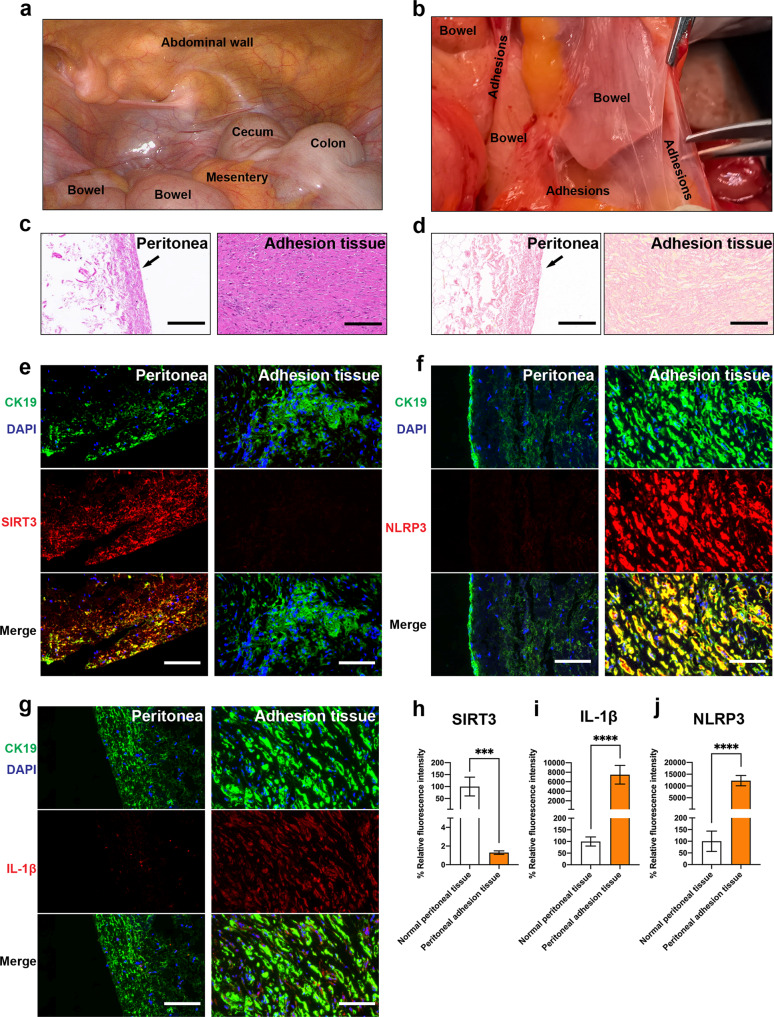
The downregulation of SIRT3 in human PAs. **a**, **b** Representative images showing normal peritoneal and adhesion tissues that were collected during laparoscopy and open surgery. **c** Representative H&E staining images showing the thickness of normal peritoneal and adhesion tissues. Scale bars: 200 µm. **d** Representative PSR staining images showing the deposition of collagen in normal peritoneal and adhesion tissues. Scale bars: 200 µm. **e** Representative immunofluorescence images showing the expression of SIRT3 (red fluorescence) in normal peritoneal and adhesion tissues; CK19 (green fluorescence) was used as a marker of mesothelial cells. Scale bars: 100 µm. **f** Representative immunofluorescence images showing the expression of NLRP3 (red fluorescence) in normal peritoneal and adhesion tissues; CK19 (green fluorescence) was used as a marker of mesothelial cells. Scale bars: 100 µm. **g** Representative immunofluorescence images showing the expression of IL-1β (red fluorescence) in normal peritoneal and adhesion tissues; CK19 (green fluorescence) was used as a marker of mesothelial cells. Scale bars: 100 µm. **h** Quantitation of the expression of SIRT3 in normal peritoneal and adhesion tissues. **i** Quantitation of the expression of NLRP3 in normal peritoneal and adhesion tissues. **j** Quantitation of the expression of IL-1β in normal peritoneal and adhesion tissues. The data are presented as the mean ± SD (*n* = 5). ***P < 0.001, ****P < 0.0001. Student’s *t*-test.

To determine whether these mechanisms of adhesion formation and SIRT3 expression observed in mice were similar to those in human tissues, normal human peritoneal tissues and adhesion tissues were obtained from patients (*n* = 10) who underwent abdominal surgeries in the Department of General Surgery of the First Affiliated Hospital of Xi’an Jiaotong University (Fig. 8a, b). Patient baseline data are shown in Supplementary Table 1. The patient tissue samples were stained with H&E to show the thickness of normal peritoneal and adhesion tissues and with PSR to show the deposition of collagen in normal peritoneal and adhesion tissues (Fig. 8c, d). To determine whether SIRT3, NLRP3, and IL-1β are mainly expressed in mesothelial cells, we performed SIRT3/CK19, NLRP3/CK19, and IL-1β/CK19 immunofluorescence staining of normal peritoneal tissues and peritoneal adhesion tissues. The results showed that NLRP3, SIRT3, and IL-1β were mainly expressed in mesothelial cells, and the expression of SIRT3 was evidently downregulated, while the expression of NLRP3 and IL-1β was upregulated in PA tissues compared with normal peritoneal tissues (Fig. 8e–j), suggesting that rescuing SIRT3 may be a therapeutic strategy to treat postsurgical PAs.

## Discussion

SIRT3 is an NAD^+^-dependent deacetylase located in mammalian mitochondria that regulate mitochondrial homeostasis under stress. In this study, we observed decreased SIRT3 expression in the PA tissues of mice and humans. Similar findings were obtained by Morigi et al. and Kurundkar et al., which showed that SIRT3 expression was reduced in cisplatin-induced AKI and lipopolysaccharide (LPS)-induced ALI^22,23^. Furthermore, we demonstrated that SIRT3 deficiency increased ROS production and mitochondrial damage, directly resulting in the activation of the NLRP3 inflammasome in PA tissues or in damaged peritoneal mesothelial cells. NLRP3, which is a key intracellular sensor, can detect a large number of pathogens, as well as some endogenous and exogenous danger signals, leading to NLRP3 inflammasome formation and activation^36^. Specifically, the NLRP3 inflammasome can be activated by several molecular and cellular events, such as calcium signaling, potassium efflux, and lysosomal damage^37^. Additionally, mitochondrial dysfunction and excess ROS have been shown to be important upstream events in NLRP3 activation. Several studies have shown that autophagy and ROS can negatively and positively regulate the NLRP3 inflammasome, respectively^38^. We sorted primary mesothelial cells from the PA tissues of wild-type and *Sirt3*^*−/−*^ mice and found that NLRP3 was strongly activated in *Sirt3*^*−/−*^ cells. Furthermore, we measured the expression of the NLRP3-related proinflammatory factors IL-1β and IL-18 and found that they were significantly upregulated in the adhesion tissues of *Sirt3*^*−/−*^ mice. To date, this is the first study describing NLRP3 activation in primary mesothelial cells, which may serve as a new therapeutic target for PAs.

Although many studies have described SIRT3 impairment in LPS-induced ALI, cisplatin-induced AKI, cardiac hypertrophy, Alzheimer’s disease, Parkinson’s disease, and cancers, we found that abdominal surgery under sterile inflammation resulted in SIRT3 deficiency in mesothelial cells^22,23,29,39–41^. SIRT3, which is one of the most dominant deacetylases in mitochondria, catalyzes the deacetylation of one-fifth of the proteins in mitochondria, such as superoxide dismutase 2 (SOD2), glutamate dehydrogenase, and acetyl-CoA synthetase 2 (AceCS2). Schwer et al. found that K642 in AceCS2 could be deacetylated by SIRT3^42^. Additionally, Pillai et al. demonstrated that SIRT3 could deacetylate SOD2 at K122 in cardiomyocytes, which enhanced its antioxidant and antiapoptotic abilities. Our results indicated that increasing the levels of SIRT3 in mesothelial cells improved their survival under H_2_O_2_-induced oxidative stress conditions.

Previous studies have revealed that the NLRP3 inflammasome plays an important role in the innate immune system, especially in bone-marrow-derived macrophages. Two classic types of signals activate the NLRP3 inflammasome: pathogen-associated molecular patterns (PAMPs) and damage-associated molecular patterns (DAMPs)^43,44^. The former includes Sendai virus, influenza virus, adenovirus, and LPS. The latter includes extracellular ATP, glucose, and amyloid. A notable result of our study was the confirmation that the NLRP3 inflammasome could be assembled and activated in mesothelial cells due to the accumulation of ROS and mitochondrial damage. Interestingly, Jia et al. also discovered that intestinal ischemia–reperfusion injury induced intestinal inflammation and activated the NLRP3 inflammasome in Caco-2 cells and intestinal epithelial cells^45^. Their research demonstrated that the administration of metformin stabilized the interaction between TXNIP and NLRP3, reducing mortality after intestinal ischemia–reperfusion injury. Based on the SIRT3 deficiency observed in PAs, we administered HKL to rescue SIRT3 expression, which decreased ROS levels and enhanced the OCR. The reduced accumulation of ROS contributed to the elimination of NLRP3 activation. Thus, the early application of HKL was beneficial for the treatment of PAs because it reduced the inflammatory process. The degree of NLRP3 activation in mesothelial cells could be used as an indicator to determine the severity of inflammation in PAs.

PAs negatively affect the quality of life of millions of people worldwide. However, surgeons do not consider adhesions to be a pressing issue, even though most of these physicians treat PAs on a daily basis. Perry et al. investigated the risk factors for PAs and reported that among 388 patients who were diagnosed with PAs, 79% had undergone previous surgery, while 18 and 3% had histories of inflammatory disease and congenital adhesions, respectively^46^. Among 955 patients undergoing laparoscopic operations, Szigetvari et al. found that severe adhesions were more common in patients with a history of abdominal or pelvic surgery than in those without such a history^47^. A history of surgery as the primary risk factor is consistent with the results of our mouse cecal abrasion postsurgical adhesion model.

A National Hospital Discharge Survey showed that between 1998 and 2002, 18.1% of annual hospitalizations were due to PAs; these hospitalizations totaled 948,000 d of inpatient care at the cost of 1.18 billion dollars. However, the specific mechanism of PA development is unclear. The latest research revealed that GATA6^+^ macrophages seal sterile operative injuries and promote the rapid repair of lesions, leading to the growth of abdominal scar tissues and the formation of PAs^48^. Of note, Tsai et al. used lineage-tracing technology and demonstrated that surgical adhesions were derived from mesothelial cells but not macrophages and suggested MLSN was a therapeutic target against PAs^49^. Furthermore, Foster et al. suggested that tissue-resident fibroblasts triggered PA and that JUN could be a therapeutic target to prevent adhesion formation^50^. In addition, berberine was shown to prevent PAs by directly inhibiting TIMP-1 expression^51^.

**Fig. 9:**
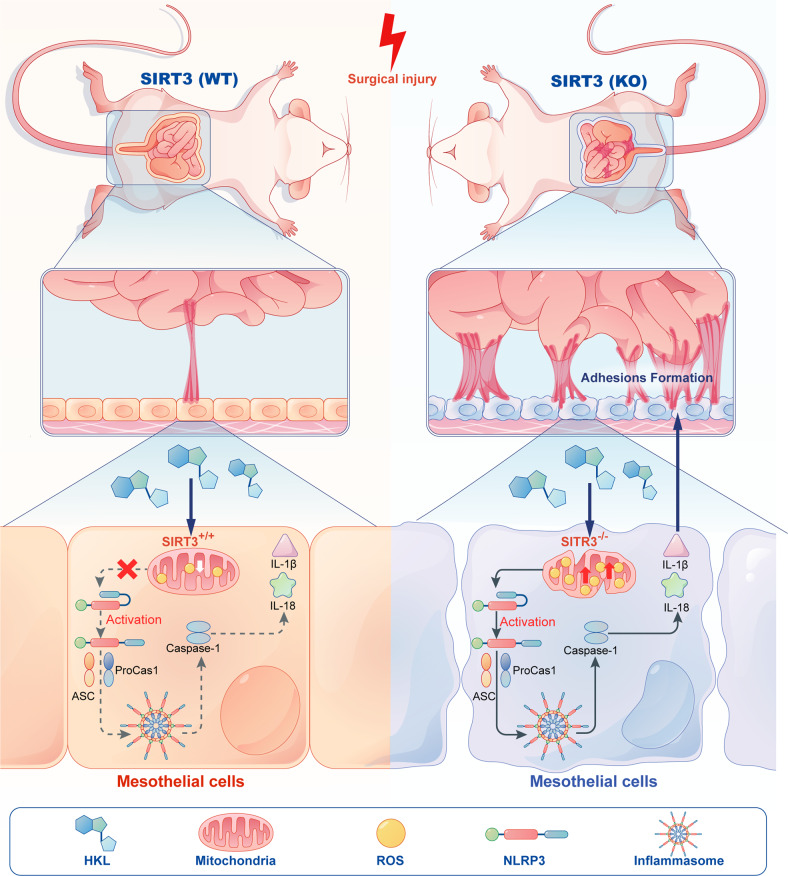
Mechanism of HKL alleviating PA. The mechanistic schematic showing that activating SIRT3 in peritoneal mesothelial cells alleviates postsurgical peritoneal adhesion formation by decreasing oxidative stress and inhibiting the NLRP3 inflammasome.

In this study, we applied HKL to treat postsurgical PAs for the first time because HKL increased SIRT3 levels in several cell types. We found that HKL increased *SIRT3* mRNA levels in primary mesothelial cells and reduced ROS formation and NLRP3-related inflammation in vitro and in vivo. Additionally, HKL clearly increased the OCR in primary mesothelial cells, which was consistent with our observation that HKL could decrease the levels of ROS in mitochondria. However, how HKL activates SIRT3 remains unclear. Gupta et al. reported that HKL can enter mitochondria to activate SIRT3, suggesting that the interaction between HKL and SIRT3 occurs in this organelle^21^. Although the mechanism by which HKL enters mitochondria has not been revealed, previous studies have shown that hydrophobic compounds such as HKL can enter mitochondria through simple diffusion. In our model of postsurgical PAs, we found that HKL reduced ROS formation, inflammation, and NLRP3-related inflammasome activation in PA tissues from wild-type mice but not *Sirt3*^*−/−*^ mice (Fig. 9). When we administered HKL to treat abdominal adhesions immediately after surgery, we found that HKL significantly reduced the thickness of the adhesions and the deposition of collagen. In contrast, delayed treatment failed to reduce adhesion severity or collagen deposition, suggesting that HKL reduces abdominal adhesion formation during the inflammation stage but not during the stage of collagen deposition. However, a few limitations in our study need to be addressed. For example, HKL only dissolves in nonpolar solvents, which restricts the methods of HKL administration. In addition, more prospective clinical studies are urgently needed to verify the effects of HKL on PA treatment.

## Supplementary information

Supplementary materials

## Data Availability

The data used to support the findings of our study are available upon reasonable request.
