# Supplementary material for: Activating SIRT3 in peritoneal mesothelial cells alleviates postsurgical peritoneal adhesion formation by decreasing oxidative stress and inhibiting the NLRP3 inflammasome
Source: Exp Mol Med. 2022 Sep 13;54(9):1486–501. doi: 10.1038/s12276-022-00848-3 (PMC9535009; doi:10.1038/s12276-022-00848-3)
Supplement: Supplementary file 1 — Supplementary materials [file 12276_2022_848_MOESM1_ESM.pdf]

Supplementary Fig. 1

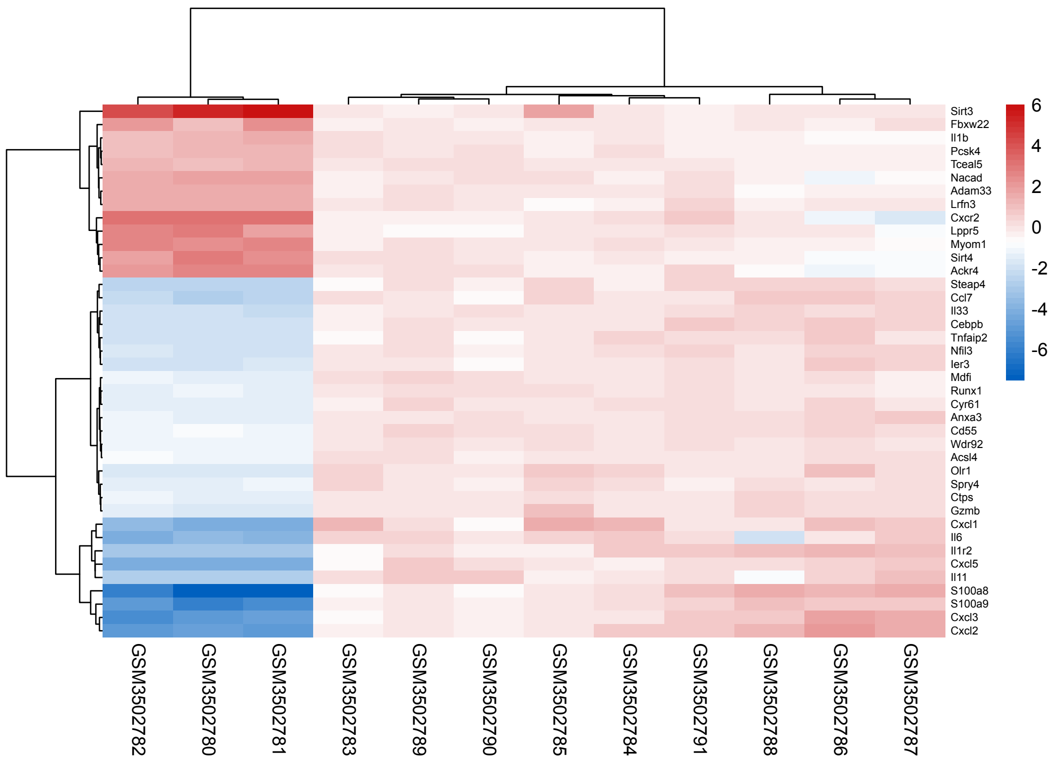

Supplementary Fig. 1 The heatmap showing the expression levels of DEGs between the postsurgical adhesion groups and the sham groups.

Supplementary Fig. 2

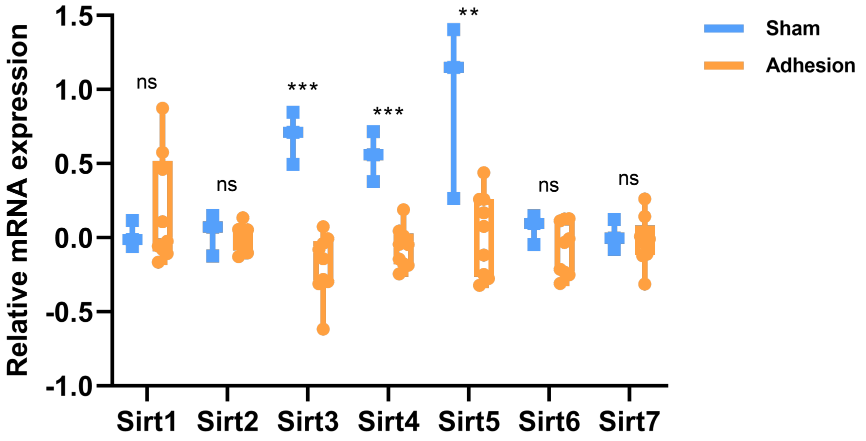

Supplementary Fig. 2 The RNA expression levels of Sirt family genes (Sirt1-7) provide by GSE123413 cohort in the sham and adhesion groups. ns, not significant; **, $P<0.01$; ***, $P<0.001$.

Supplementary Fig. 3

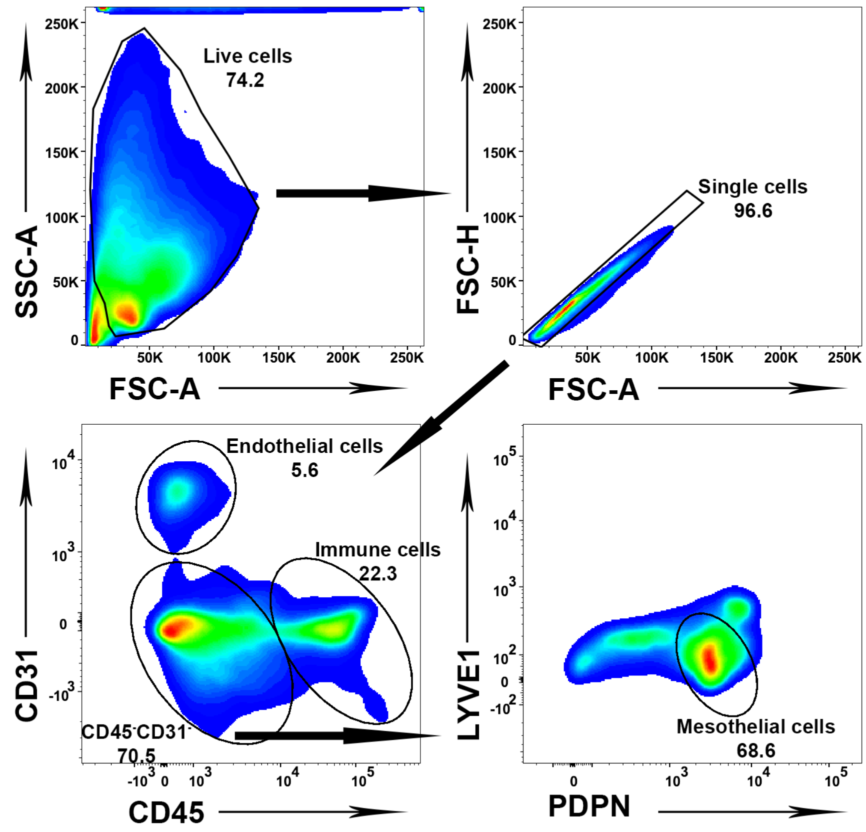

Supplementary Fig. 3 Gating strategy for mesothelial cells, immune cells and endothelial cells in adhesion tissues by flow cytometry.

Supplementary Fig. 4

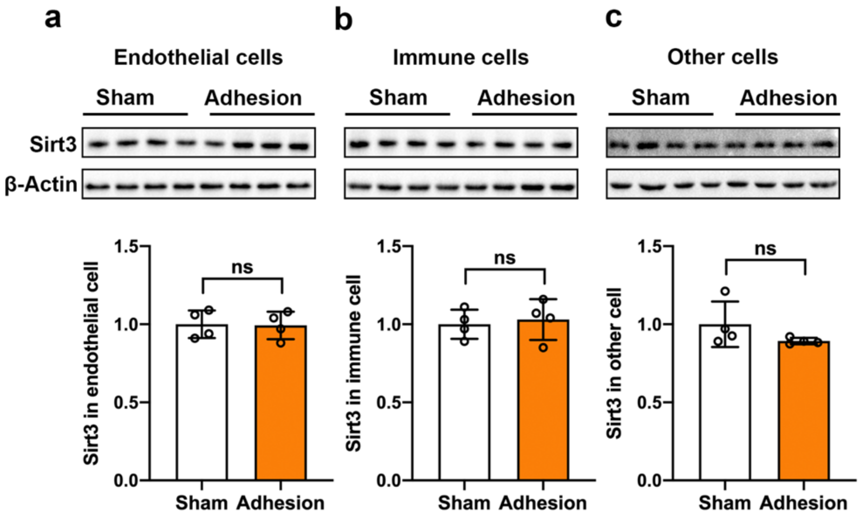

Supplementary Fig. 4 Representative Western blots of SIRT3 expression in the endothelial cells (a), immune cells (b) and other cells (c) from sham and adhesion tissues. The data are presented as the mean ± SD (n = 4). ns, not significant. Student's t test. All experiments were repeated three times.

Supplementary Fig. 5

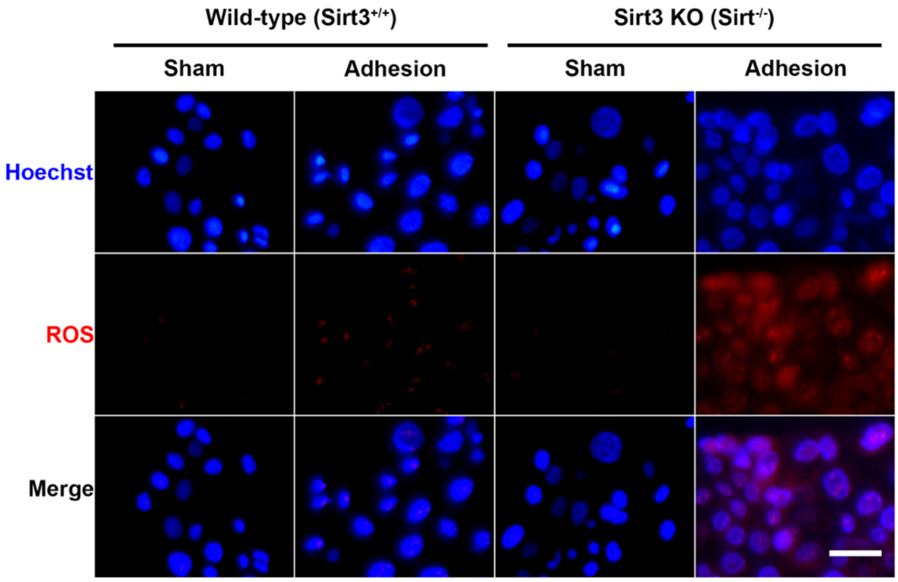

Supplementary Fig. 5 Representative images of ROS accumulation (red fluorescence) in sorted primary mesothelial cells from wild-type and *Sirt3*$^{-/-}$ mice. Scale bar: 50 µm.

Supplementary Fig. 6

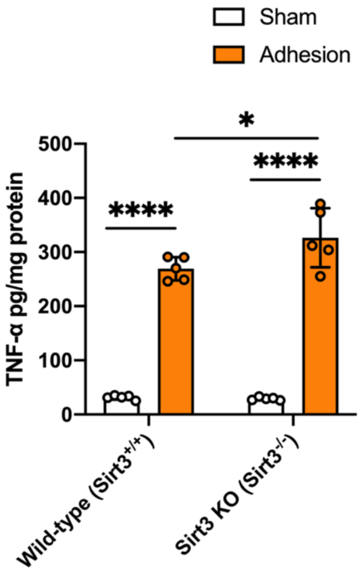

Supplementary Fig. 6 The levels of TNF-α in wild-type and *Sirt3*$^{-/-}$ adhesion tissues were measured by ELISA. The data are presented as the mean ± SD (n = 5). *P<0.05, ****P < 0.0001. Two-way ANOVA. All experiments were repeated three times.

Supplementary Fig. 7

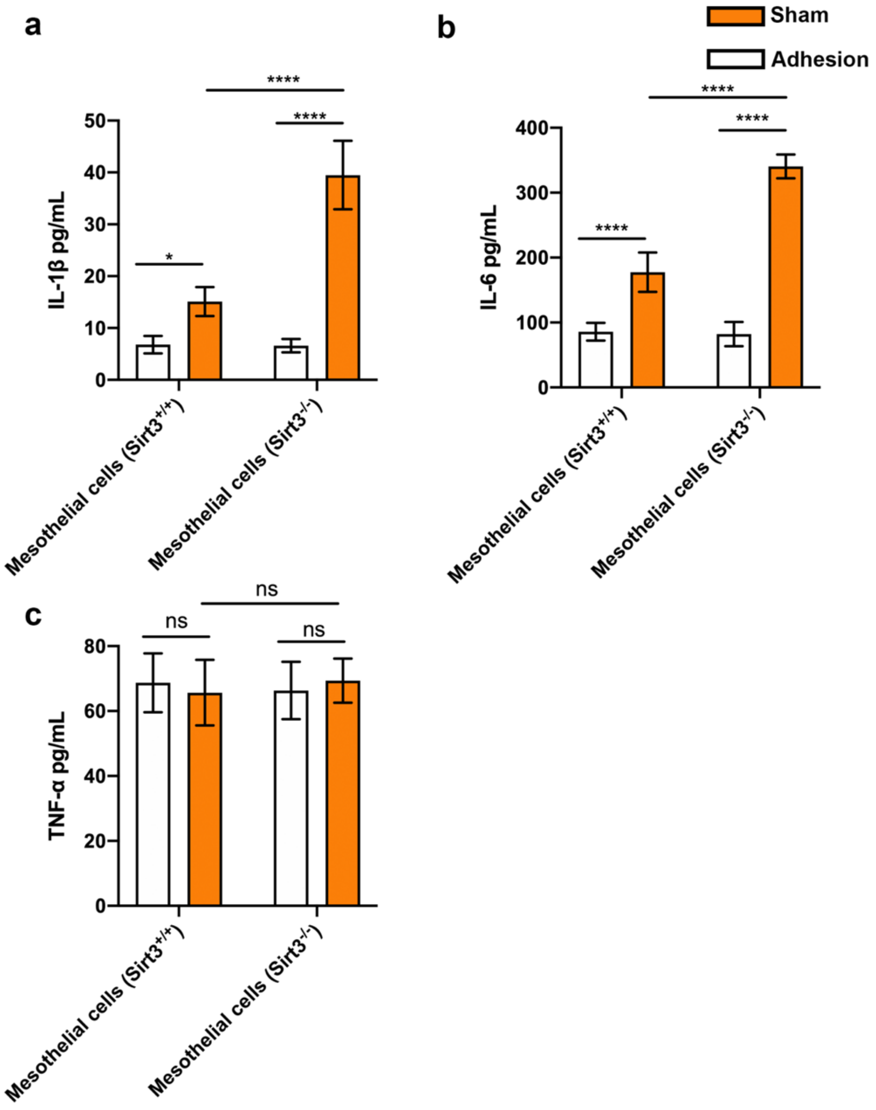

Supplementary Fig. 7 The levels of IL-1β, IL-6 and TNF-α in sham and adhesion primary mesothelial cells from wild-type and *Sirt3*$^{-/-}$ mice were measured by ELISA. The data are presented as the mean ± SD (n = 5). ns, not significant, **P*<0.05, *****P* < 0.0001. Two-way ANOVA. All experiments were repeated three times.

Supplementary Fig. 8

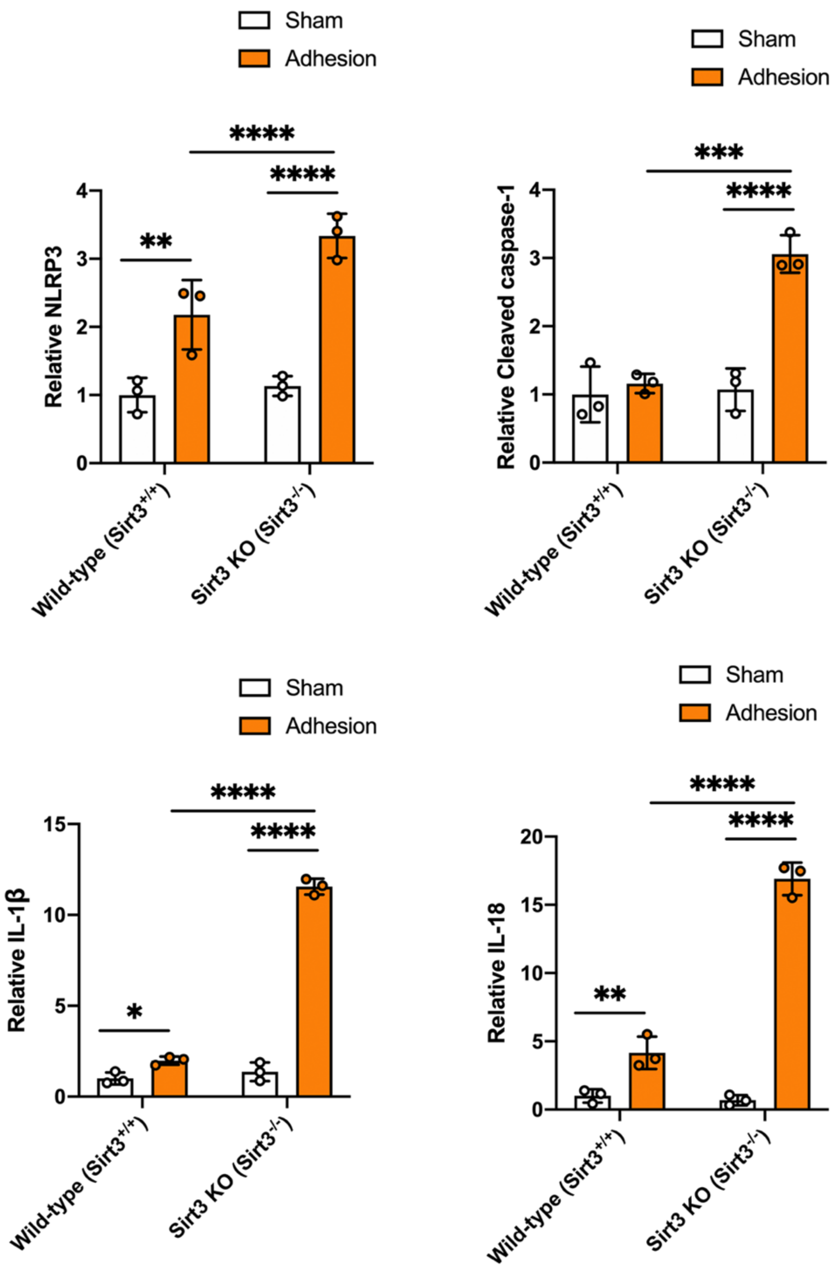

Supplementary Fig. 8 Quantitation of Western blots of NLRP3, cleaved caspase-1, IL-1β and IL-18 in sham and adhesion tissues from wild-type and *Sirt3*$^{-/-}$ mice. The data are presented as the mean ± SD (n = 5). **P*<0.05, ***P*<0.01, ****P*<0.001, *****P* < 0.0001. Two-way ANOVA. All experiments were repeated three times.

Supplementary Fig. 9

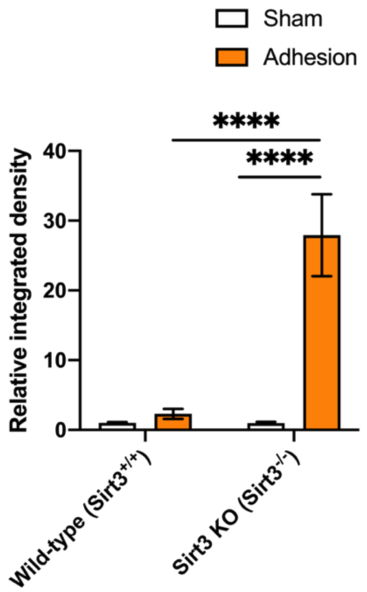

Supplementary Fig. 9 Quantitation of the expression of NLRP3 (red fluorescence) in sham and adhesion primary mesothelial cells from wild-type and *Sirt3*$^{-/-}$ mice. The data are presented as the mean ± SD (n = 4). ****P < 0.0001. Two-way ANOVA. All experiments were repeated three times.

Supplementary Fig. 10

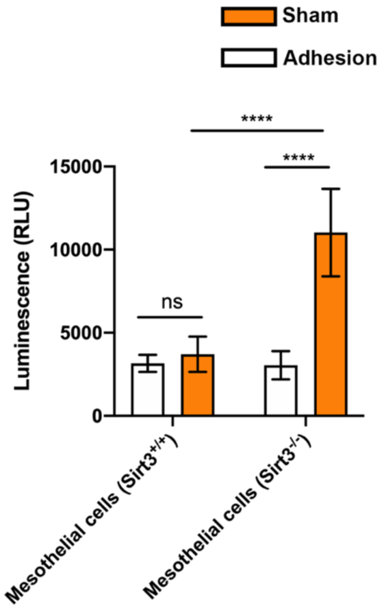

Supplementary Fig. 10 The activity of caspase-1 in sham and adhesion primary mesothelial cells from wild-type and *Sirt3*$^{-/-}$ mice were measured by ELISA. The data are presented as the mean ± SD (n = 5). ns, not significant, ****P < 0.0001. Two-way ANOVA. All experiments were repeated three times.

Supplementary Fig. 11

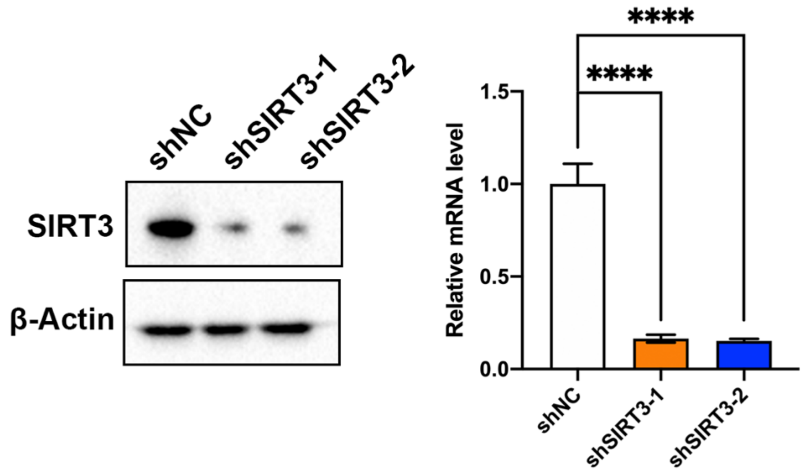

Supplementary Fig. 11
Western blot and PCR detection of SIRT3 protein and mRNA expression levels in shNC, shSIRT3-1 and shSIRT3-2 MeT-5A cells. The data are presented as the mean ± SD (n = 3). ****P < 0.0001. One-way ANOVA. All experiments were repeated three times.

Supplementary Fig. 12

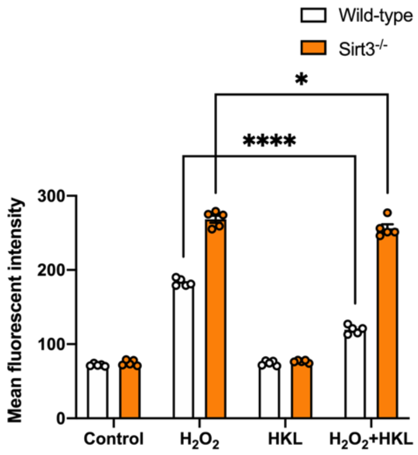

Supplementary Fig. 12 Quantitation of the levels of ROS in wild-type and *Sirt3*$^{-/-}$ mesothelial cells treated with H_2O_2 and/or HKL. The data are presented as the mean ± SD (n = 5). *P < 0.05, ****P < 0.0001. Two-way ANOVA. All experiments were repeated three times.

Supplementary Fig. 13

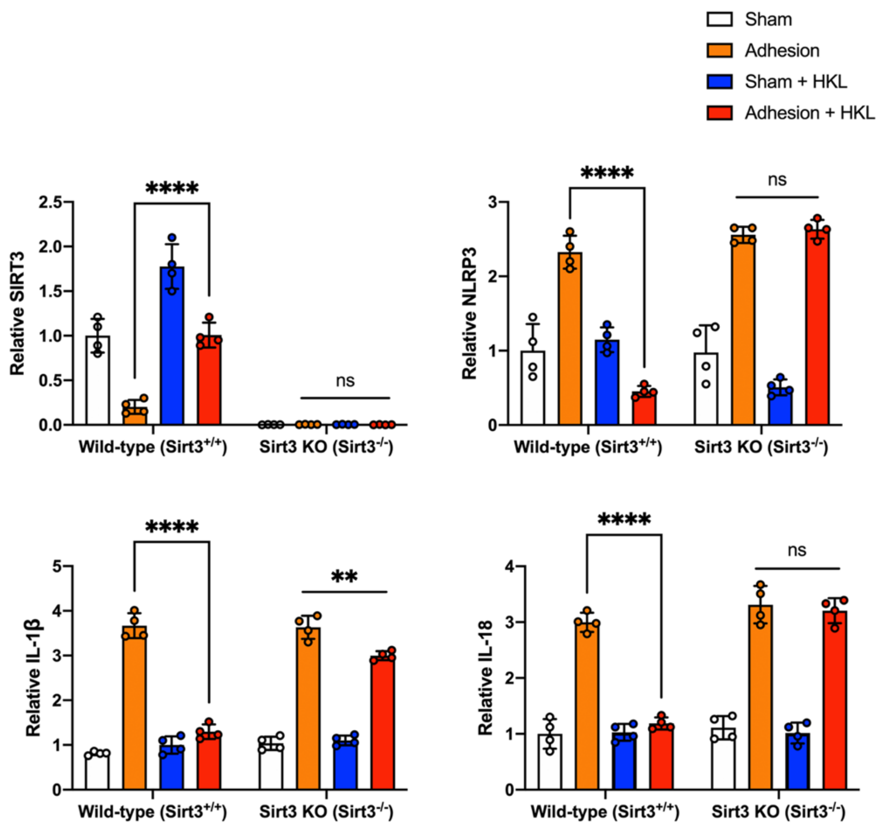

Supplementary Fig. 13 Quantitation of Western blots of SIRT3, NLRP3, IL-1β and IL-18 in sham and adhesion tissues from wild-type and *Sirt3*$^{-/-}$ mice treated with Intralipid or HKL. The data are presented as the mean ± SD (n = 4). ns, not significant, ***P* < 0.01, *****P* < 0.0001. Two-way ANOVA. All experiments were repeated three times.

Supplementary Fig. 14

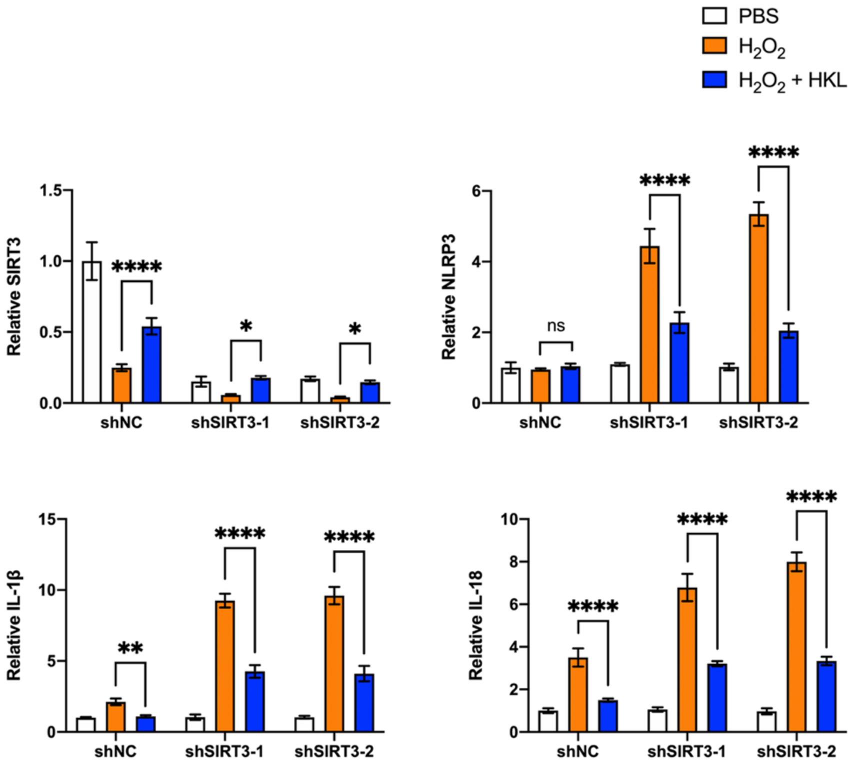

Supplementary Fig. 14 Quantitation of Western blots of SIRT3, NLRP3, IL-1β and IL-18 in shNC, shSIRT3-1 and shSIRT3-2 MeT-5A cells treated with H_2O_2 and/or HKL. The data are presented as the mean ± SD (n = 3). ns, not significant, **P* < 0.05, ***P* < 0.01, *****P* < 0.0001. Two-way ANOVA. All experiments were repeated three times.

Supplementary Table 1. Normal and adhesion patient baseline data.

Patient ID	Adhesion State	Gender	Age	Diagnosis
1701619	Adhesion	Male	56	Post-LC adhesions
1502231	Adhesion	Male	48	Post-LC adhesions
1701441	Adhesion	Female	62	Post-LC adhesions
1762512	Adhesion	Female	31	Post- appendectomy adhesions
1309678	Adhesion	Male	62	Post-LC adhesions
1730312	Normal	Male	38	Hernia
1700921	Normal	Male	28	Hernia
1467822	Normal	Male	42	Hernia
1792124	Normal	Male	30	Hernia
1701369	Normal	Male	36	Hernia
